# Normal Human Gingival Epithelial Cells Sense *C. parapsilosis* by Toll-Like Receptors and Module Its Pathogenesis through Antimicrobial Peptides and Proinflammatory Cytokines

**DOI:** 10.1155/2010/940383

**Published:** 2010-05-03

**Authors:** Raouf Bahri, Sèverine Curt, Dalila Saidane-Mosbahi, Mahmoud Rouabhia

**Affiliations:** ^1^Groupe de Recherche en Écologie Buccale, Faculté de Médecine Dentaire, Pavillon de Médecine Dentaire, Université Laval, QC, Canada G1K 7P4; ^2^Laboratoire d'Analyse, Traitement et Valorisation des Polluants de l'Environnement et des Produits, Faculté de Pharmacie, Rue Avicenne, 5019 Monastir, Tunisia

## Abstract

This study was designed to investigate the interaction between *C. parapsilosis* and human epithelial cells using monolayer cultures and an engineered human oral mucosa (EHOM). *C. parapsilosis* was able to adhere to gingival epithelial cells and to adopt the hyphal form in the presence of serum. Interestingly, when cultured onto the engineered human oral mucosa (EHOM), *C. parapsilosis* formed small biofilm and invaded the connective tissue. Following contact with *C. parapsilosis*, normal human gingival epithelial cells expressed high levels of Toll-like receptors (TLR)-2, -4, and -6, but not TLR-9 mRNA. The upregulation of TLRs was paralleled by an increase of IL-1*β*, TNF*α*, and IFN*γ* mRNA expression, suggesting the involvement of these cytokines in the defense against infection with *C. parapsilosis*. The active role of epithelial cells in the innate immunity against *C. parapsilosis* infection was enhanced by their capacity to express high levels of human beta-defensin-1, -2, and -3. The upregulation of proinflammatory cytokines and antimicrobial peptide expression may explain the growth inhibition of *C. parapsilosis* by the gingival epithelial cells. Overall results provide additional evidence of the involvement of epithelial cells in the innate immunity against *C. parapsilosis* infections.

## 1. Introduction

Fungal infections caused by *Candida* species are increasing, particularly in immunocompromized individuals [1]. *C. albicans* is the most common opportunistic fungal pathogen involved in health problems encountered in humans. However, infections by other *Candida* species are gaining ground worldwide, particularly in nosocomial settings. These emerging candidiases involve various *Candida* species, including *C. tropicalis*, *C. krusei*, *C. glabrata*, and *C. parapsilosis* [2]. 

In comparison to other *Candida* species, *C. parapsilosis* is largely distributed in nature. Unlike *C. albicans* and *C. tropicalis, C. parapsilosis* is not an obligate human pathogen, having been isolated from nonhuman sources [3] such as domestic animals, insects, soil, and marine environments [4]. *C. parapsilosis* is also a normal human commensal, and it is one of the fungi most frequently isolated from the subungal space of human hands. *C. parapsilosis* is a commensal of human skin, and its pathogenicity is limited by intact integument. *C. parapsilosis* is notorious for its capacity to grow in total parenteral nutrition and to form biofilms on catheters and other implanted devices, for nosocomial spread by hand carriage, and for persistence in clinical units [5]. *C. parapsilosis* is of special concern in critically ill neonates leading to high morbidity and mortality [6]. Furthermore, C. parapsilosis has been isolated from the oral cavity of of HIV-infected [7] and diabetic individuals [8]. Although these studies document *C. parapsilosis* pathogenicity, further investigations are warranted to determine how human tissues can interact with, and prevent *C. parapsilosis* infection. 

It is well known that the first line of defense against exogenous microorganism infections lies in the tissues, such as the skin and the oral mucosa [9, 10]. Both of these tissues are layered with an epithelial structure containing over 95% epithelial cells that not only have an active defensive role as a mechanical barrier but are also key players in antimicrobial innate immunity [7, 10]. The attachment of microorganisms such as *C. parapsilosis* to host cells is the initial stage of the infection process, enabling *candida* to survive and eventually colonize the host tissue during the development of candidiasis. Recognition of adherent *C. parapsilosis* by epithelial cells may involve specific receptors, including Toll-like receptors (TLRs) which play an active role by recognizing pathogens of considerable target specificity [11]. This recognition leads to a series of signaling events that result in the necessary acute host responses required to kill the pathogens [12]. Recent studies have demonstrated the involvement of TLRs in the recognition of fungal pathogens such as *C. albicans*, although less is known regarding the function of these receptors following *Candida* infection [13, 14]. The *Candida*-killing activity of keratinocytes involves TLR-2 and TLR-4 through NF-*κ*B activation [15]. The host's response to infection is most likely due to the different recognition/activation patterns of these receptors and the release of several proinflammatory cytokines [16, 17].

In response to *C. albicans* infection, oral epithelial cells were shown to produce significant amounts of IL-6, IL-8, and TNF*α* [18, 19], which suggests that cytokines have a significant role in controlling oral infections. Controlling *Candida* pathogenesis also involves the defensin family (human *β*-defensins, HBDs) of antimicrobial peptides that display broad-spectrum antimicrobial activity against a large array of pathogens, such as *Candida* species [20]. Expressed by different cell types, including epithelial cells, these peptides have been suggested as playing an active role in the commensal-to-pathogenic microorganism activity in epithelial cells [21]. While the antifungal activity of HBDs has been characterized to some extent [22], their exact role in the host's defense mechanism against fungal infection remains unclear. Recent studies showed that the salivary levels of HBD-1 and -2 were lower in patients with oral candidiasis than in healthy individuals [23], whereas HBD-2 peptide expression was greater in human oral epithelia with candidiasis than in normal oral epithelia [24].

Given that *C. parapsilosis* can be acquired from different sites including soil and marine environments, and that *C. parapsilosis* is considered as an emerging pathogen in humans, we proposed to study (1) the virulence of *C. parapsilosis* (transition from blastospore to hyphal form) when cultured under the appropriate conditions (neutral pH in the presence or absence of serum), and its susceptibility to an antifungal agent (amphotericin B), (2) the ability of *C. parapsilosis* to attach to normal human epithelial cells in a monolayer culture and to form biofilm when used to infect EHOM in vitro, and (3) epithelial cell defenses against *C. parapsilosis* infection by investigating the gene activation of TLRs, antimicrobial peptides (HBD-1, -2, -3, and -4), and pro-inflammatory cytokines (IL-1*β*, TNF*α*, and IFN*γ*). 

## 2. Materials and Methods

### 2.1. *Candida* Species

*C. parapsilosis* was isolated from water and sediment samples taken from various sites in the Mediterranean Sea near the city of Gabes, Tunisia (North Africa). The collected samples were immediately stored on ice and transported to the laboratory. To isolate and identify *C. parapsilosis*, a suspension of 50 g of sediment from each sediment sample was prepared in 100 mL of saline solution. The water samples were used undiluted. Both the sediment suspensions (1 mL) and the water samples (1 mL) were laid over Sabouraud dextrose agar (Difco; Becton-Dickinson, Sparks, MD) enriched with yeast extract and were incubated at 30°C [25]. Following microorganism growth, separate colonies were resuspended and seeded on Sabouraud dextrose agar gel and incubated at 30°C for 24 hours. To obtain homogenous yeast suspensions, separate colonies were used to inoculate different tubes containing Sabouraud dextrose broth (Difco; Becton-Dickinson) supplemented with 0.1% glucose and antibiotics (streptomycin/penicillin at 20 *μ*g/mL) to inhibit potential bacterial growth. The yeasts were grown to the stationary phase overnight at 30°C in a shaking water bath. Blastoconidia were then collected, washed with PBS, and subjected to specific identification. *C. parapsilosis* was selected following a rapid identification method based on the colorimetric detection of carbon assimilation (Api ID 32C, Biomérieux SA, Marcy l'Étoile, France). This identification step was performed three times using *Candida* subculture from the same single colony used for the initial identification test. We were thus able to confirm that the *Candida* species involved was indeed *C. parapsilosis*. In some tests, we used *C. albicans* (ATCC 10231) as a reference species. Following cultures, *C. parapsilosis* and *C. albicans* were collected, washed with phosphate-buffered saline (PBS), counted using a hemocytometer [9], adjusted to 10^7^/mL, and set aside for testing.

### 2.2. *C. parapsilosis* Susceptibility to Amphotericin-B

*C. parapsilosis* (10^4^/well) was seeded in a round-bottom 96-well plate with or without various concentrations of amphotericin-B (1,000-0 ng/mL). The *Candida* cells in the presence or absence of varied concentrations of antifungal agent were incubated at 37°C for 24 hours. *C. parapsilosis* susceptibility to amphotericin-B was compared to that of *C. albicans* (ATCC-10231). *Candida* growth was assessed using the MTT (3-(4,5-dimethylthiazol-2-yl)-2,5-diphenyl tetrazolium bromide) assay, which measures cell growth as a function of mitochondrial activity [18]. Standard growth curves were used to determine the concentrations of viable *C. parapsilosis* and *C. albicans* following treatment with amphotericin-B and the minimum inhibitory concentration (MIC), referring to the lowest concentration of amphotericin-B leading to a total inhibition of *Candida* growth.

### 2.3. *C. parapsilosis* Virulence through the Blastospore-Hyphal Transition

The transition from blastospore to hyphal form is considered to be a virulence factor that enables *Candida* to deeply penetrate the tissue and infect the host. We therefore investigated the capacity of isolated *C. parapsilosis* to adopt the hyphal form under favorable conditions. It has been shown that temperature, proteins, and neutral pH promote *Candida* transition [26]. We therefore cultured *C. parapsilosis* under three different conditions to assess the presence/absence of hyphae. *C. albicans* (ATCC 10231) was used as a reference. The germ tube formation of both *Candida* strains was examined in the presence of 10% FCS, at neutral pH (6 and 7), and finally at 30°C and 37°C. To do so, *Candida* (10^6^ cells) was grown in 2 mL of Sabouraud dextrose broth under one of the above mentioned conditions for 3 and 6 hours. At the end of each culture period, the *Candida* cultures were observed microscopically, and the germ tubes and total cells were counted and used to determine the transition percentage [18]. 

### 2.4. *C. parapsilosis* Adherence and Growth following Contact with Gingival Epithelial Cells

#### 2.4.1. Isolation and Culture of Oral Mucosal Cells

Small pieces of palatal mucosa were biopsied from gingival graft patients following their informed consent and with the approval of the Université Laval Ethical Committee. The epithelium was separated from the lamina propria using thermolysin treatment (500 *μ*g/mL), after which time the epithelial cells were isolated with a 0.05% trypsin-0.01 EDTA solution. We used 0.125 U/mL of collagenase-P (Boehringer Mannheim, Laval, Québec, Canada) to extract fibroblasts from the lamina propria. Oral epithelial cells (9 × 10^3^ cells/cm^2^) were cultured with 1.3 × 10^4^/cm^2^ irradiated mouse 3T3 fibroblasts in Dulbecco's modified Eagle-Ham's F_12_ (3 : 1) (DMEM) supplemented medium [27]. Oral fibroblasts (1 × 10^4^ cells/cm^2^) were grown in Dulbecco's modified Eagle (DME) supplemented medium. When the cultures reached 70%–80% confluence for the epithelial cells and 100% for the fibroblasts, the cells were detached and used for various tests.

#### 2.4.2. Contact between *Candida* and Epithelial Cells

Cells were seeded in six-well tissue culture plates and grown at 37°C in an 8% CO_2_ atmosphere. At 80% confluence, epithelial cell monolayers were pulsed with *C. parapsilosis* (10^5^ cells/cm^2^) for 3, 6, 12, and 24 hours. At the end of each contact period, the monolayers were used to assess *C. parapsilosis* adhesion and growth. To assess *C. parapsilosis* adhesion, monolayer cultures were washed six times with PBS and fixed with 40% methanol and 60% acetone for 10 minutes. The cells were washed twice with PBS and stained with crystal violet. The stained monolayers were then examined under an optical microscope and photographed. To assess the growth of *C. parapsilosis* following contact with the epithelial cells, culture medium was used to collect nonadherent *C. parapsilosis* as well as those adhering to the cells. This collection was performed by treating the monolayer epithelial cells with 1% (v/v) Triton X-100 in PBS to lyse the epithelial cells with no side effect on the *Candida*. A pellet of *C. parapsilosis* collected from the culture medium was then mixed with those adhering to and entering the epithelial cells. *C. parapsilosis* viability and numbers under each condition were determined by trypan blue dye exclusion [9].

#### 2.4.3. Effect of *C. parapsilosis* on Epithelial Cell Viability

Immediately after each contact period, cells were detached from the culture flasks using a 0.05% trypsin-0.01 M EDTA solution for 20 minutes at 37°C. The cells were washed twice with DMEM-supplemented culture medium. The pellet was resuspended in 500 *μ*L of DMEM and 50 *μ*L of each epithelial cell suspension were withdrawn to assess the percentage of viable cells using the trypan blue exclusion technique [19].

### 2.5. Effect of *C. parapsilosis* on Tissue Structure

#### 2.5.1. Preparation of Engineered Human Oral Mucosa

Using normal primary oral epithelial cells and oral fibroblasts, we produced engineered human oral mucosa (EHOM) [28]. Briefly, oral fibroblasts were mixed with bovine skin collagen (BD Biosciences, Mississauga, ON, Canada) to produce the lamina propria. Tissue was grown in Dulbecco-Vogt modified Eagle's medium supplemented with 5% fetal calf serum for 4 days. The oral epithelial cells were then seeded onto the lamina propria, and the tissue was grown in the presence of 10% fetal calf serum. Once the epithelial cells reached confluence, the EHOM were raised to the air-liquid interface for 5 days to enable the epithelium to stratify. Following this air-liquid interface culture, the EHOM were used to study the tissue interaction of *C. parapsilosis*.

#### 2.5.2. Tissue Structure following *C. parapsilosis* Infection

To test the effect of *C. parapsilosis* on tissue structure, the EHOM tissue was infected with 1 × 10^6^ cells/cm^2^ of *C. parapsilosis*. Tissue untreated with *Candida* cells served as the control. Following a 24-h infection period, the tissue was collected and used for histological analyses. We collected multiple biopsies from each infected EHOM and stained them with hematoxylin and eosin. Each slide was assigned a number and blindly analyzed for tissue structure and the presence of *C. parapsilosis* at regular intervals using a calibrated image analysis system [29].

### 2.6. Effect of *C. parapsilosis* on the Expression of TLRs, *β*-Defensins, and Pro-Inflammatory Cytokines by Gingival Epithelial Cells

#### 2.6.1. Stimulation of Epithelial Cells with *C. parapsilosis*

Epithelial cells were detached from 75-cm^2^ culture flasks using trypsin, washed twice in culture medium, counted, seeded into six-well tissue culture plates (Falcon, Becton Dickinson, Lincoln Park, NJ) at 2.5 × 10^5^ cells/well, and finally incubated in an 8% CO_2_ atmosphere at 37°C. After 5 days of culture, the epithelial cells reached approximately 80% confluence. They were then stimulated or not with *C. parapsilosis* (10^5^/cm^2^) and were cultured again for 6 and 24 hours. Total RNA was extracted from the cells following each culture period.

#### 2.6.2. RNA Extraction and Quantification

Total cellular RNA content was extracted using the Illustra RNAspin Mini (GE Health Care UK Limited, Buckingham, UK), while RNA concentration, purity, and quality were determined by means of the Experion system and RNA StdSens analysis kit according to instructions provided by the manufacturer (Bio-Rad, Hercules, CA).

#### 2.6.3. Quantitative Real-Time RT-PCR

RNA (1 *μ*g of each sample) was reverse transcripted into cDNA using Maloney murine leukemia virus (M-MLV) reverse transcriptase (Invitrogen Life Technologies, Mississauga, ON, Canada) and random hexamers (Amersham Pharmacia Biotech, Inc., Baie d'Urfé, QC, Canada). RT conditions were 10 minutes at 65°C, 1 hour at 37°C, and 10 minutes at 65°C. Quantitative PCR was carried out as previously described [18]. Amounts of mRNA transcripts were measured using the Bio-Rad CFX96 real-time PCR detection system (Bio-Rad, Mississauga, ON, Canada). Reactions were performed using a PCR supermix from Bio-Rad (iQ SYBR Green supermix). Primers (Table 1) were added to the reaction mix at a final concentration of 250 nM. Five microliters of each cDNA sample was added to a 20 *μ*L PCR mixture containing 12.5 *μ*L of iQ SYBR Green supermix (Bio-Rad) and 0.5 *μ*L of specific primers (TLR-2, TLR-4, TLR-6, TLR-9, HBD-1, HBD-2, HBD-3, HBD-4, GAPDH, IL-1*β*, TNF*α*, and IFN*γ*) (Medicorp, Inc., Montréal, QC, Canada) and 7 *μ*L of RNase and DNase free water (MP Biomedicals, Solon, OH). Each reaction was performed in a Bio-Rad MyCycler Thermal Cycler (Bio-Rad). For the qPCR, the CT was automatically determined using the accompanying Bio-Rad CFX manager. Thermocycling conditions for the TLRs were 5 minutes at 95°C, followed by 45 cycles of 95°C for 15 seconds, and 60°C for 30 seconds and 72°C for 30 seconds, with each reaction done in triplicate. For the HBDs, the thermocycling conditions were 95°C for 3 minutes, followed by 45 cycles of 95°C for 10 seconds, 63°C for 10 seconds, and 72°C for 30 seconds, with each reaction performed in triplicate. For the cytokines, the conditions were 95°C for 10 minutes, followed by 40 cycles of 95°C for 15 seconds, 60°C for 60 seconds, and 72°C for 30 seconds, with each reaction done in triplicate. The specificity of each primer pair was verified by the presence of a single melting temperature peak. GAPDH produced uniform expression levels varying by less than 0.5 CTs between sample conditions and was therefore used as a reference gene for this study.

### 2.7. Measurement of Antimicrobial Peptides Secretion following Tissue Contact with *C. parapsilosis*

To quantify human *β*-defensins hBD2 and hBD3, culture supernatants obtained from duplicate or triplicate experiments were analyzed by sandwich enzyme-linked immunosorbent assays (ELISA). Once the collection of supernatants was cleared by centrifugation, HBD2 and HBD3 were measured in triplicate using an ELISA kit (Phoenix Pharmaceuticals Inc., Burlingame, California, USA) according to the manufacturer's instructions. To do so, 50 *μ*L volumes of undiluted supernatants were used to perform the quantitative analyses. Following incubation, the plates were read at 450 nm and analyzed using a Microplate Reader Model 680 (Bio-Rad Laboratories Ltd, Mississauga, ON). The experiments were repeated three times and the means ± standard deviation (SD) were presented.

### 2.8. Statistical Analyses

Experimental values are given as means ± SD. The statistical significance of differences between the control values and the test values was determined using a one-way ANOVA. Posteriori comparisons were done using Tukey's method. Normality and variance assumptions were verified using the Shapiro-Wilk test and the Brown and Forsythe test, respectively. All of the assumptions were fulfilled. *P*-values were declared significant at 0.05. Data were analyzed using the SAS version 8.2 statistical package (SAS Institute, Inc., Cary, NC).

## 3. Results

### 3.1. Susceptibility of *C. parapsilosis* to Amphotericin-B

**Figure 1:**
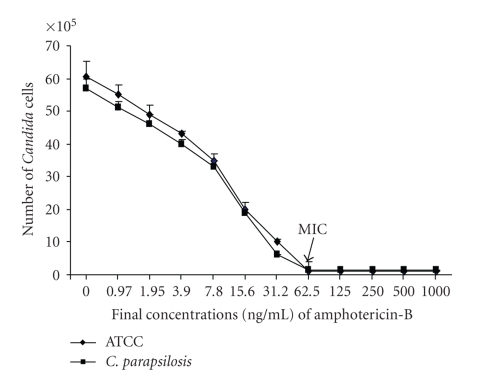
Sensitivity of *C. parapsilosis* to amphotericin-B. The yeast was cultured in the presence of various concentrations of amphotericin-B for 24 hours and its growth was assessed by MTT assay. Quantification of live *C. parapsilosis* was obtained using a standard live curve. The effect of the antifungal agent on *C. parapsilosis* was compared to that on *C. albicans*. Results are presented as means ± SD of six different experiments.

*Candida* strains were isolated from Mediterranean Sea water and sediment samples. Api ID 32C analysis revealed that *C. parapsilosis* was the most isolated strain. Following the extraction and identification of *C. parapsilosis*, we were concerned about its possible side effects on humans; we therefore tested its susceptibility to an antifungal agent. Figure 1 shows that the growth rate of *C. parapsilosis* was very slow compared to that of *C. albicans* (ATCC 10231). In the presence of amphotericin B, the MIC was in the rage of 60 ng/mL for both C. albicans and *C. parapsilosis*, which suggests that *C. parapsilosis* was as sensitive to amphotericin B as *C. albicans*.

### 3.2. Isolated *C. parapsilosis* Was Able to Adopt the Filamentous Form

**Figure 2:**
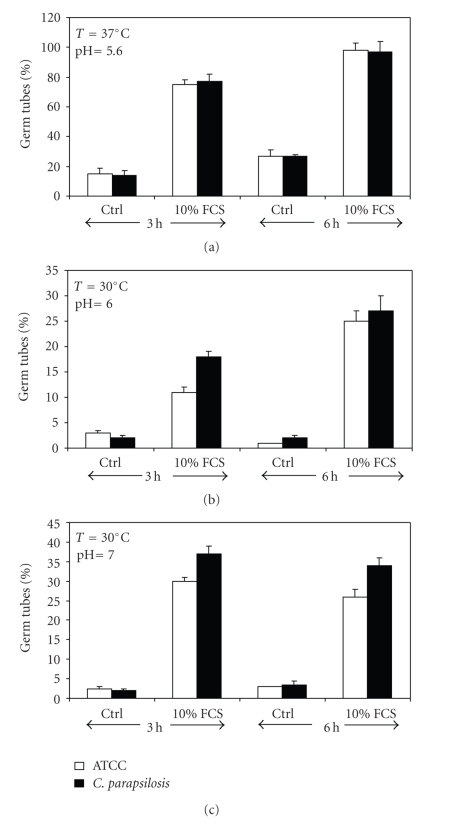
Blastospore-to-hyphal transition of *C. parapsilosis*. Following culture at different temperatures and neutral pH with or without proteins (serum), the percentage of *C. parapsilosis* germ tubes was determined by dividing the number of hyphae over the total number of cells (blastospore and hyphae) in each culture condition. The mean relative values for six separate experiments are shown. *C. albicans* was used as a reference strain to *C. parapsilosis*.

As the phenotype change (i.e., transition from yeast to filamentous form) of *Candida* is one of the major determinants of its virulence and is closely associated with its ability to cause disease, we investigated *C. parapsilosis* transition under various culture conditions. As shown in Figure 2, both *C. albicans* and *C. parapsilosis* were able to adopt the filamentous form under any favorable conditions. Indeed, at neutral pH with 10% serum, *C. parapsilosis* displayed filamentous forms after 3 and 6 hours of culture. The same observations were obtained at acidic pH in the presence of serum.

### 3.3. *C. parapsilosis* Adhered to Gingival Epithelial Cells

**Figure 3:**
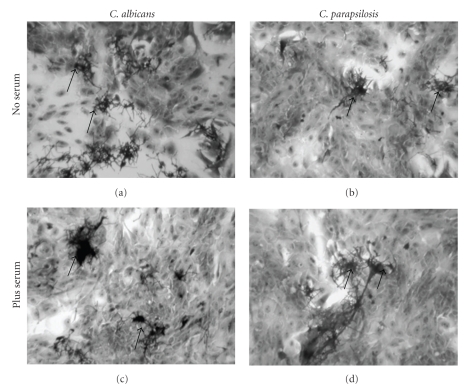
*C. parapsilosis* adhesion to the gingival epithelial monolayer culture. Human gingival cells were grown up to 80% confluence, pulsed with *C. parapsilosis* or *C. albicans*, and incubated for 6 hours with or without serum. Following this culture period, the supernatant containing nonattached *Candida* was discarded, and the cultures were washed and stained with Cristal violet. Representative photographs are presented. Magnification: 250×.

Adhesion of *C. parapsilosis* to epithelial cells was investigated at 6 and 24 hours postinfection. As shown in Figure 3, different clamps of *C. parapsilosis* were present on the epithelial cell monolayer 6 hours after infection and were distributed throughout the culture. Of great interest was that in the presence of serum, these clamps were bigger than were those without serum. Also, with serum, morphological changes (hyphae forms) were more visible as compared to culture without serum. The same results were obtained at 24 hours postinfection (data not shown). *C. parapsilosis* thus adhered to normal human gingival epithelial cells and adopt filamentous phenotype, which may explain its pathogenic capacity in humans.

### 3.4. Effect of Human Gingival Epithelial Cells on *C. parapsilosis* Growth

As epithelial cells downregulate *C. albicans* growth [9], and because *C. parapsilosis* adheres to gingival epithelial cells, we investigated the effect of epithelial cells on the growth of *C. parapsilosis*. Table 2 shows that following contact with epithelial cells, all of the collected *C. parapsilosis* were alive, yet their numbers were significantly reduced, varying from 1.5 × 10^5^/cm^2^ after a 3-h contact to approximately 3 × 10^3^/cm^2^ after a 24-h contact. These results suggest that gingival epithelial cells inhibited the growth of *C. parapsilosis*. It is interesting to note that to some extent, epithelial cells killed off *C. parapsilosis* because at the longer contact period (24 hours), there were far less (3 × 10^3^/cm^2^) *C. parapsilosis* present than what was initially used (10^5^/cm^2^) to infect the epithelial cell cultures. On the other hand, epithelial cell viability following contact with C. parapsilosis was investigated showing that (Table 2) no significant differences were obtained between *C. parapsilosis*-infected and uninfected cultures, either in terms of cell viability or total numbers of viable cells.

### 3.5. *C. parapsilosis* Formed a Biofilm on the Engineered Human Oral Mucosa Tissue Which May Have Contributed to Tissue Disorganization

**Figure 4:**
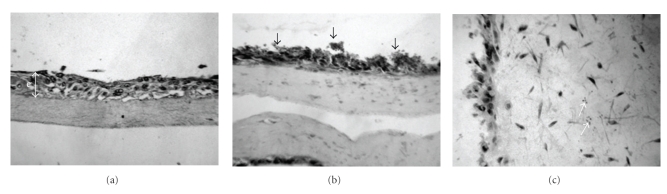
Effect of *C. parapsilosis* on tissue structure and the formation of a biofilm on the engineered human oral mucosa. Histological features of EHOM following exposure to *C. parapsilosis*. EHOM tissue was infected with (a) no *Candida* cells (uninfected control) and (b, c) *C. parapsilosis* strain. Note the formation of a biofilm on the tissue (b) and the presence of *C. parapsilosis* in the connective tissue (c). Representative photographs of four different experiments are shown (two EHOMs per experiment). Magnification ×250 for parts (a) and (b), and ×600 for part (c). e: epithelium.

To direct our studies closer to the in vivo setting, we examined the ability of *C. parapsilosis* to form biofilm on the in vivo-like EHOM model, which mimics the physiologic conditions and complex structure of the oral mucosa in terms of three-dimensional structure, relationship between the various cell types, and secretion of soluble factors [28]. Our results reveal that *C. parapsilosis* adhered to and formed biofilms on the EHOM layers (Figure 4(b), arrows). More importantly, *C. parapsilosis* did penetrate the tissue to reach the lamina propria of the EHOM, therefore, showing an invasion of the tissue layers (Figure 4(c), arrows). This invasion by *C. parapsilosis* may explain the disorganization observed in the infected tissue (Figure 4(b)) compared to the noninfected tissue (Figure 4(a)). Indeed, the tissue disorganization was characterized by differentiated cells (large cells, faint nuclei, large cytoplasm) in the upper layers of the epithelium, and the absence of layers including the stratum corneum. These studies show that *C. parapsilosis* was able to form a biofilm on the tissue to deeply invade the connective tissue, which contributed to damaging the structure of the mucosal tissue. 

### 3.6. *C. parapsilosis* Modulated TLR Expression by Normal Human Gingival Epithelial Cells

**Figure 5:**
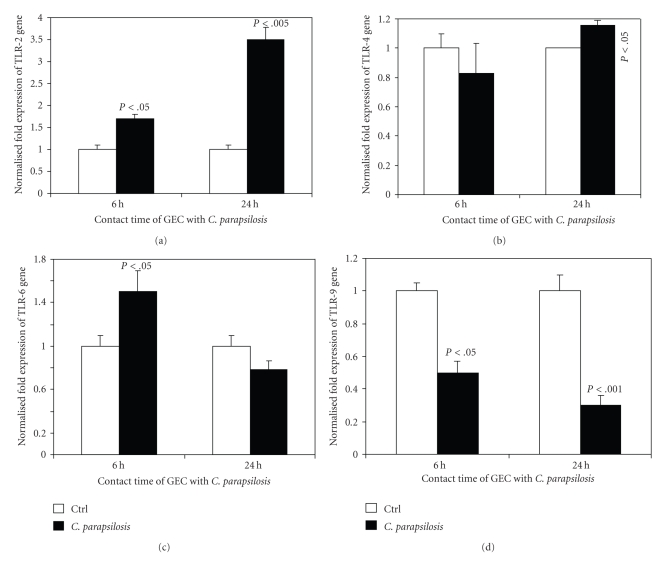
Quantification of mRNA expression levels of TLR-2, -4, -6, and -9 by gingival epithelial cells following infection with *C. parapsilosis*. Gingival epithelial cells were cultured with or without *C. parapsilosis* for 6 and 24 hours. Total RNA content was extracted from the cells following each culture period and was used for quantitative RT-PCR of the TLR-2, -4, -6, and -9 genes, as described in Section 2. Results are presented as a fold expression of the gene in the test sample compared to this gene expression in the control. Data are expressed as means ± SD from triplicate assays of three different experiments. (a) TLR-2 expression, (b) TLR-4 expression, (c) TLR-6 expression, and (d) TLR-9 expression. GEC: gingival epithelial cells.

Oral mucosa represents a major interaction site between the host and the environment. The host's line of defense at this interface is therefore critical to controlling infection. Epithelial cells are involved in the control of *C. albicans* pathogenesis through different pathways, including specific receptors such as TLRs [11, 17]. As epithelial cells downregulate *C. parapsilosis* growth, we sought to investigate the involvement of TLR expression by epithelial cells in the recognition of *C. parapsilosis*. Using quantitative RT-PCR, we showed (Figure 5(a)) that *C. parapsilosis* significantly increased TLR-2 mARN expression, at 6 (*P* < .05) and 24 hours (*P* < .005) of contact. It should be noted that the epithelial cells in contact with *C. parapsilosis* expressed more TLR-2 mRNA at 24 hours than at 6 hours (Figure 5(a)). Following TLR-2 activation, we then examined TLR-4 expression by the gingival epithelial cells. As demonstrated in Figure 5(b), the cells expressed high levels of TLR-4 mRNA later (24 hours), in response to *C. parapsilosis*. The TLR-6 mRNA expression (Figure 5(c)) showed that *C. parapsilosis* induced a significant increase of TLR-6 mRNA expression by epithelial cells at 6 hours of culture but not at 24 hours. Finally, *C. parapsilosis* modulated TLR-9 expression differently. As shown in Figure 5(d), *C. parapsilosis* significantly (*P* < .05, *P* < .001) reduced TLR-9 gene expression. Overall results suggest that gingival epithelial cells involve TLR-2, -4, and -6, but not TLR-9 in the defense against *C. parapsilosis* infections. 

### 3.7. Human Gingival Epithelial Cells Prevented *C. parapsilosis* Infection through *β*-Defensin Expression (HBD-1, HBD-2, and HBD-3)

**Figure 6:**
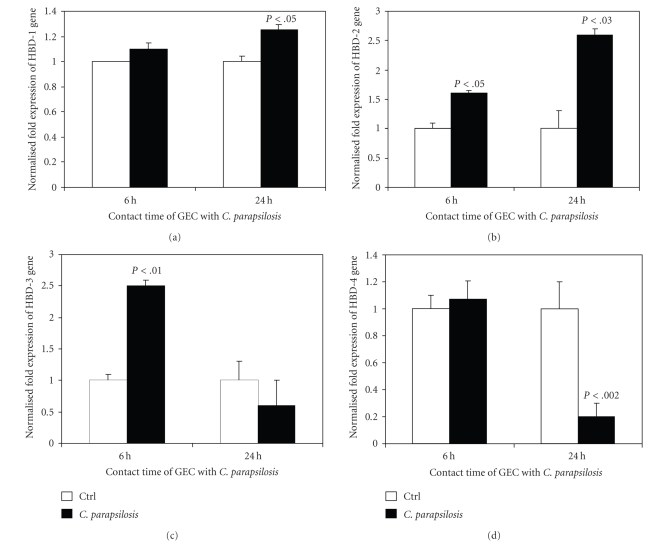
Quantification of mRNA expression levels of HBD-1, -2, -3, and -4 by gingival epithelial cells following infection with *C. parapsilosis*. Gingival epithelial cells were cultured with or without *C. parapsilosis* for 6 and 24 hours. Total RNA was extracted from the cells following each culture period and was used for quantitative RT-PCR of the HBD-1, -2, -3, and -4 genes, as described in Section 2. Results are presented as a fold expression of the gene in the test sample compared to this gene expression in the control. Data are expressed as means ± SD from triplicate assays of three different experiments. (a) HBD-1 expression, (b) HBD-2 expression, (c) HBD-3 expression, and (d) HBD-4 expression. GEC: gingival epithelial cells.

**Figure 7:**
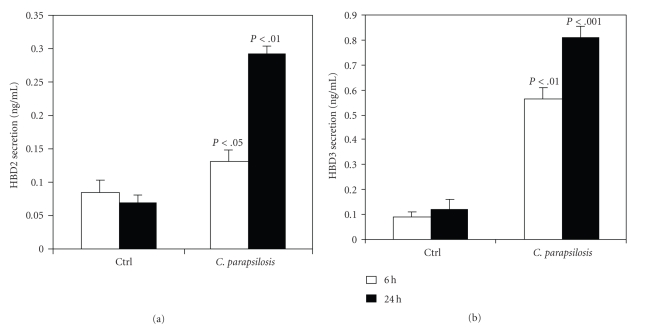
Quantification of HBD2 and HBD3 levels secreted by gingival epithelial cells following infection with *C. parapsilosis*. Gingival epithelial cells were cultured with or without *C. parapsilosis* for 6 and 24 hours. Supernatants were collected and used to quantify HBD2 (a) and HBD3 (b) using ELISA kits. Data are means ± SD of three separate experiments. The levels of significance were obtained by comparing the amount of AMP obtained with unstimulated samples to that obtained with samples stimulated *C. parapsilosis. *

Antimicrobial peptides (AMP) are a component of mammalian innate immunity and are expressed by various cell types to prevent microbial infection. It is well known that epithelial cells involve defensins to control bacterial infection through TLRs. As we revealed that TLR expression was modulated by *C. parapsilosis* (Figure 5), we also examined the expression of HBD-1, HBD-2, HBD-3, and HBD-4 in response to stimulation with *C. parapsilosis*. As reported in Figure 6(a), *C. parapsilosis* significantly (*P* < .05) increased HBD-1 mRNA expression by epithelial cells at 24 hours. We also showed (Figure 6(b)) a significant increase of HBD-2 at 6 hours (*P* < .05) and 24 hours (*P* < .03) following cell contact with *C. parapsilosis*. Epithelial cells expressed elevated HBD-3 mRNA levels at 6 but not at 24 hours in response to *C. parapsilosis* stimulation (Figure 6(c)). However, HBD-4 mRNA remained unchanged at the early contact period but decreased at the longer contact period (24 hours) (Figure 6(d)). These results suggest that gingival epithelial cells control *C. parapsilosis* pathogenesis through antimicrobial peptides, particularly HBD-1, HBD-2, and HBD-3. To confirm these results, we analysed the secretion of HBD2 and HBD3 following *C. parapsilosis* infection. As showed in Figure 7, the basal level of HBD2 and HBD3 increased following tissue infection with *C. parapsilosis*. This supports the increased mRNA gene expression of AMP by epithelial cells in response to *C. parapsilosis*.

### 3.8. Gingival Epithelial Cells Expressed High Levels of Proinflammatory Cytokines against *C. parapsilosis*

**Figure 8:**
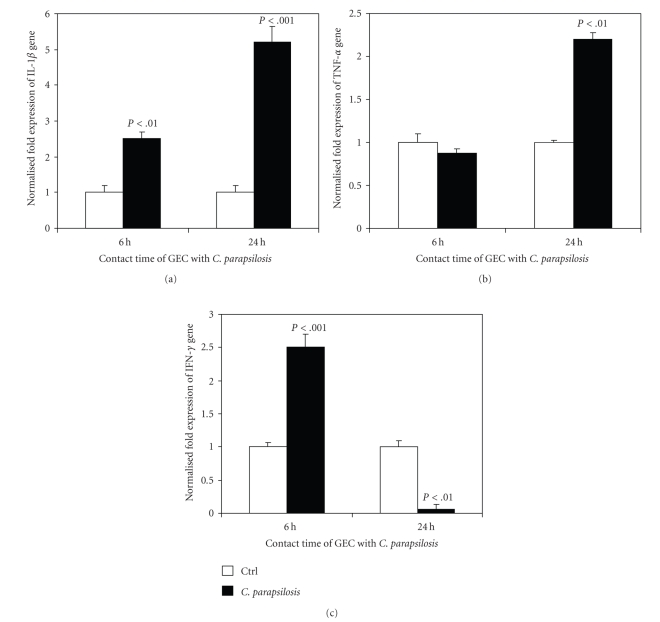
Quantification of mRNA expression levels of IL-1*β*, TNF*α*, and IFN*γ* by gingival epithelial cells following infection with *C. parapsilosis*. Gingival epithelial cells were cultured with or without *C. parapsilosis* for 6 and 24 hours. Total RNA was extracted from the cells following each culture period and was used for quantitative RT-PCR of the IL-1*β*, TNF*α*, and IFN*γ* genes, as described in Section 2. Results are presented as a fold expression of the gene in the test sample compared to this gene expression in the control. Data are expressed as means ± SD from triplicate assays of three different experiments. (a) IL-1*β* expression, (b) TNF*α* expression, and (c) IFN*γ* expression. GEC: gingival epithelial cells.

Cytokine proteins IL-1*β*, TNF*α*, and IFN*γ* are active in controlling oral infections and maintaining the symbiotic relationship between the oral microbial community and the host. We therefore examined the gene expression of these interleukins by gingival epithelial cells following contact with *C. parapsilosis*. As shown in Figure 8(a), epithelial cells expressed a high level of IL-1*β* mRNA in response to *C. parapsilosis*. This elevated expression was present at 6 and 24 hours postinfection but was not time-dependent. *C. parapsilosis* also stimulated the epithelial cells to express a high level of TNF*α* mRNA basically after 24 hours of stimulation (Figure 8(b)). On the other hand, *C. parapsilosis* significantly (*P* < .001) increased the IFN*γ* mARN expression by the gingival epithelial cells at early (6 hours) contact period, but decreased IFN*γ* mRNA expression at 24 hours postinfection. Overall results suggest that gingival oral epithelial cells prevented *C. parapsilosis*'s side effect through pro-inflammatory cytokines including IL-1*β*, IFN*γ*, and TNF*α*.

## 4. Discussion

It is well documented that non*albicans Candida* (NAC) species cause high levels of human candidiasis [2] which is more prevalent in immunocompromized patients. The most involved NAC species in human disease are *C. tropicalis*, *C. krusei*, and *C. glabrata* [30, 31]. To these we add another emerging species, *C. parapsilosis*, responsible for multiple human candidiasis cases [32]. In comparison to other *Candida* species, *C. parapsilosis* has an extensive distribution in nature. Unlike *C. albicans* and *C. tropicalis, C. parapsilosis* is not an obligate human pathogen, having been isolated from nonhuman sources [3] such as domestic animals, insects, soil, and marine environments [4]. This raises the question as to whether humans may be contaminated by *C*. *parapsilosis*. Based on available data, the answer to this question may reside in the interaction of humans with various environmental sites such as rivers, recreational locations (beaches), or through marine products. Indeed, this study showed the presence of *C. parapsilosis* in sea water and sediments. It also confirms previously reported data [33] showing the presence of different *Candida* species (including *C*. *parapsilosis*) in marine sites. As *C. parapsilosis* may reach humans via various exposures, we investigated the susceptibility of the yeast to antifungal agent amphotericin-B. Interestingly, we found that *C. parapsilosis* isolated from sea water and sediment was sensitive to this antifungal agent, thus confirming previous data [32]. Although isolated *C. parapsilosis* was sensitive to the antifungal agents, we pursued further to investigate its interaction with host cells such as normal human gingival epithelial cells. Our results indicate that isolated *C. parapsilosis* adhered to gingival epithelial cells, and adapted filamentous form. However, the hyphae from was not well expressed as the one observed with *C. albicans*, which is in accordance with previously reported data [33, 34]. However, even if it did not translate from blastospore to clear hyphal form, *C. parapsilosis* was able to reach the connective tissue of the infected EHOM. This invasion may be due to (1) the capacity of some *C. parapsilosis* cells to overcome the epithelial cells' innate defenses or (2) the large number of *C. parapsilosis* cells (10^6^/cm^2^ of tissue) used in our experiments. This large number may have overwhelmed the capacity of the epithelial structure to prevent *C. parapsilosis* invasion. Thus, further studies are required to shed light on the possible mechanisms used by *C. parapsilosis* to invade gingival tissue.

The adhesion and interaction mechanisms between *C. parapsilosis* and epithelial cells are not well known, and most of the available data corresponds to studies performed specifically with *C. albicans* [9, 10]. According to these studies, *C. parapsilosis* sensing by human epithelial cells may be through specific receptors such as TLRs. In light of this, our study confirms an increase of TLR-2, TLR-4, and TLR-6, but not TLR-9 mARN expression following interaction between *C. parapsilosis* and human epithelial cells. Although ours is the first study with *C. parapsilosis*, it confirms the involvement of the TLR (-2, -4, and -6) pathway in the interaction between host and other *Candida* species including *C. albicans* [10, 17, 35]. The nonactivation of TLR-9 mRNA expression by *C. parapsilosis* is surprising, as TLR-9 reportedly plays an active role in the recognition of *C. albicans* [36]. The recognition of *C. albicans* and *C. parapsilosis* by epithelial cells appears to involve TLR-9 in a different manner. This may constitute an experimental clinical marker to discriminate between *C. albicans* and *C. parapsilosis* infections. Further studies will provide insight on this possible pathway. Additional research is also required to shed light on the role of each of the TLR2, 4, 6, and 9 in controlling *C. parapsilosis* infection, and how the pro-inflammatory response is triggered and by which part of *C. parapsilosis*.

It is well known that TLRs play a critical role as they recognize pathogens and activate the adaptive immune response [35]. In this study, epithelial cells, another key member of the innate immunity network, expressed various inflammatory mediators (IL-1*β*, TNF*α*, and IFN*γ*) following contact with *C. parapsilosis*. These findings suggest that epithelial cells support the host's defenses against *C. parapsilosis* through proinflammatory cytokines, which is in agreement with previously reported data involving *C. albicans* [19, 37, 38]. Proinflammatory cytokines IL-1*β* and TNF*α* were augmented by the *C. parapsilosis*-infected oral epithelial cells at early and late infection periods, while IFN*γ* was involved only at early defense phase, as previously reported with *C. albicans* [9]. This study therefore confirms the capacity of oral epithelial cells, through the expression of IL-*β*, TNF*α*, and IFN*γ*, to control *C. parapsilosis* infection.

In vitro studies have shown the stimulatory effect of IFN*γ* on the phagocytosis and elimination of *C. albicans* by neutrophils and macrophages [39, 40]. In accordance with these data, we found that *C. parapsilosis* growth was reduced following culture in the presence of oral epithelial cell (Table 2). This situation may mimic the physiological and pathological environment in the oral cavity, and supports previously reported studies related to the growth inhibition of *Candida* by epithelial cells [9]. This growth inhibition may be achieved through the contribution of antimicrobial peptides. Indeed, our study demonstrates that following infection with *C. parapsilosis*, gingival epithelial cells expressed high levels of HBD-1, HBD-2, and HBD-3, which confirms the antifungal activity of *β*-defensins against *C. albicans* [41], and suggests the involvement of these antimicrobial peptides in controlling *C. parapsilosis* growth/pathogenesis by epithelial cells.

It is important to note that HBD-4 not only appeared to be unrequired but even decreased in this antifungal defense, suggesting the presence of different pathways involving HBD-4 separate from the other HBDs. Our results on HBD-4 confirm those previously reported showing a different effect of HBD-4, depending on the microorganism used [42]. Indeed, HBD-4 has been shown to display weak antimicrobial activity against *E. coli*, *S. cerevisiae*, *S. aureus*, *S. pneumoniae*, and *B. cepacea*, and strong antimicrobial activity against *S. carnosus* and *P. aeruginosa* [43]. The effect of HBD-4 remains to be elucidated basically following infection with *Candida*.

## 5. Conclusion

This study demonstrated some key events related to the interaction between gingival epithelial cell/tissues and *C. parapsilosis*. Through this interaction, gingival epithelial cells involved TLRs and different mediators to control *C. parapsilosis* pathogenesis. Additional research will nevertheless be necessary to fully determine the key events involved in host/*C. parapsilosis* interactions and the mechanisms that prevent *C. parapsilosis* infection. Epithelial cells could be instrumental in helping us unravel the opportunistic process by which *C. parapsilosis*, found in proximity to humans, becomes a pathogen.

## Figures and Tables

**Table 1:** Primer sequences used for the q-RT-PCR.

Gene name	Primers sequences	Product size (bp)
TLR-2	sense: 5′-GCCTCTCCAAGGAAGAATCC-3′	144
	antisense: 5′-TCCTGTTGTTGGACAGGTCA-3′	

TLR-4	sense: 5′-AATCTAGAGCACTTGGACCTTTCC-3′	116
	antisense: 5′-GGGTTCAGGGACAGGTCTAAAGA-3′	

TLR-6	sense: 5′-CATCCTATTGTGAGTTTCAGGCAT-3′	121
	antisense: 5′-GCTTCATAGCACTACATCCCAAG-3′	

TLR-9	sense: 5′-GGACCTCTGGTACTGCTTCCA-3′	151
	antisense: 5′-AAGCTCGTTGTACACCCAGTCT-3′	

*β*1-defensin	sense: 5′-GCCTCTCCCCAGTTCCTGAA-3′	82
	antisense: 5′-GCAGAGAGTAAACAGCAGAAGGTA-3′	

*β*2-defensin	sense: 5′-TGTGGTCTCCCTGGAACAAAAT-3′	105
	antisense: 5′-GTCGCACGTCTCTGATGAGG-3′	

*β*3-defensin	sense: 5′-CTTCTGTTTGCTTTGCTCTTCCT-3′	138
	antisense: 5′-CTGTTCCTCCTTTGGAAGGCA-3′	

*β*4-defensin	sense: 5′-CACTCTACCAACACGCACCTAG-3′	133
	antisense: 5′-CGCAACTGGAACCACACACT-3′	

IL-1*β*	sense: 5′-CTGTCCTGCGTGTTGAAAGA-3′	69
	antisense: 5′-TTGGGTAATTTTTGGGATCTACA-3′	

IFN*γ*	sense: 5′-GGCATTTTGAAGAATTGGAAAG-3′	111
	antisense: 5′-TTTGGATGCTCTGGTCATCTT-3′	

TNF*α*	sense: 5′-CAGCCTCTTCTCCTTCCTGAT-3′	122
	antisense: 5′-GCCAGAGGGCTGATTAGAGA-3′	

GAPDH	sense: 5′-GGTATCGTCGAAGGACTCATGAC-3′	180
	antisense: 5′-ATGCCAGTGAGCTTCCCGTTCAGC-3′	

**Table 2:** Growth of *C. parapsilosis *following contact with human oral epithelial cells, and viability of epithelial cells after contact with *Candida*.

Contact time (h)	Nb. of *C. parapsilosis * ^&^ (×10^5^/cm^2^)	Viability of *epithelial cells *(%)
3	1.6 ± 0.7	97.3 ± 0.01
6	0.9 ± 0.03**	97.0 ± 0.02
12	0.1 ± 0.09**	96.5 ± 0.32
24	0.03 ± 0.01**	96.9 ± 0.37

^&^ *C* *. parapsilosis* was seeded onto epithelial cell culture at 10^5^ cells/cm^2^. To assess the growth of *Candida* following contact with the epithelial cells, nonadherent (in the supernatant) and adherent (on the monolayer epithelial cell cultures) *C. parapsilosis* were collected and enumerated. To assess the viability of epithelial cells, cultures were treated with trypsin and cells were washed twice then viable epithelial cells were determined using trypan bleu exclusion test.

**P ≤ .01 is level of significance for growth inhibition of *C. parapsilosis *in infected oral epithelial cell cultures compared to initial seeding concentration of *C. parapsilosis.*
